# Structure and dynamics of a human myelin protein P2 portal region mutant indicate opening of the β barrel in fatty acid binding proteins

**DOI:** 10.1186/s12900-018-0087-2

**Published:** 2018-06-25

**Authors:** Saara Laulumaa, Tuomo Nieminen, Arne Raasakka, Oda C. Krokengen, Anushik Safaryan, Erik I. Hallin, Guillaume Brysbaert, Marc F. Lensink, Salla Ruskamo, Ilpo Vattulainen, Petri Kursula

**Affiliations:** 1 Faculty of Biochemistry and Molecular Medicine, University of Oulu, Oulu, Finland; 2 European Spallation Source (ESS), Lund, Sweden; 3 Department of Physics, Tampere University of Technology, Tampere, Finland; 4 Department of Biomedicine, University of Bergen, Bergen, Norway; 5 Unité de Glycobiologie Structurale et Fonctionnelle, University of Lille, CNRS UMR8576 UGSF, F-59000 Lille, France; 6 Department of Physics, University of Helsinki, Helsinki, Finland

**Keywords:** Myelin, Crystal structure, Fatty acid-binding protein, Membrane binding, Molecular dynamics, Mutation, Protein stability

## Abstract

**Background:**

Myelin is a multilayered proteolipid sheath wrapped around selected axons in the nervous system. Its constituent proteins play major roles in forming of the highly regular membrane structure. P2 is a myelin-specific protein of the fatty acid binding protein (FABP) superfamily, which is able to stack lipid bilayers together, and it is a target for mutations in the human inherited neuropathy Charcot-Marie-Tooth disease. A conserved residue that has been proposed to participate in membrane and fatty acid binding and conformational changes in FABPs is Phe57. This residue is thought to be a gatekeeper for the opening of the portal region upon ligand entry and egress.

**Results:**

We performed a structural characterization of the F57A mutant of human P2. The mutant protein was crystallized in three crystal forms, all of which showed changes in the portal region and helix α2. In addition, the behaviour of the mutant protein upon lipid bilayer binding suggested more unfolding than previously observed for wild-type P2. On the other hand, membrane binding rendered F57A heat-stable, similarly to wild-type P2. Atomistic molecular dynamics simulations showed opening of the side of the discontinuous β barrel, giving important indications on the mechanism of portal region opening and ligand entry into FABPs. The results suggest a central role for Phe57 in regulating the opening of the portal region in human P2 and other FABPs, and the F57A mutation disturbs dynamic cross-correlation networks in the portal region of P2.

**Conclusions:**

Overall, the F57A variant presents similar properties to the P2 patient mutations recently linked to Charcot-Marie-Tooth disease. Our results identify Phe57 as a residue regulating conformational changes that may accompany membrane surface binding and ligand exchange in P2 and other FABPs.

**Electronic supplementary material:**

The online version of this article (10.1186/s12900-018-0087-2) contains supplementary material, which is available to authorized users.

## Background

A multitude of proteins interact with lipid membrane surfaces. These peripheral membrane proteins come from various protein families, and have generally little in common at the structural level. Both electrostatic and hydrophobic interactions are important for protein binding onto membranes. Conformational changes in the protein are often observed upon membrane binding and embedding, and the dynamics of both the protein and lipid components may be altered upon proteolipid complex formation. The proteins of the myelin sheath are in intimate contact with lipid membranes, being either peripheral or integral membrane proteins [1]. Defects in myelin protein structure and/or function are linked to various chronic demyelinating conditions, such as multiple sclerosis and peripheral neuropathies.

Peripheral myelin protein P2 is one of the most abundant proteins in human peripheral nervous system (PNS) myelin [2]. P2 is expressed inhomogeneously along myelinated axons: it can reach 15% of total myelin protein in selected areas in the PNS, and it is also expressed in the central nervous system in small amounts in the spinal cord and brain stem [2–4]. The physiological role of P2 remains unclear, even though it is expressed at such high levels in myelin. P2 is located in compact myelin, and it is expressed more in large axon myelin [3, 5]. P2 has the ability to stack membrane leaflets together [6, 7], and a dysfunction of P2 may lead to myelin degeneration. Recently, three point mutations in human P2 were linked to Charcot-Marie-Tooth disease (CMT), an inherited neuropathy [8–10]. We showed that these mutations all decrease the stability of P2, although they did not lead to major differences in the P2 fold in the crystal state [11]. The disease mutations also slightly altered membrane binding properties of P2 [11].

The structure of human P2 has been solved at 0.93-Å resolution using X-ray crystallography [12]. P2 belongs to the fatty acid binding protein (FABP) family, and the structure of P2 is typical for FABPs [13, 14]: the body of P2 is a barrel formed of 10 antiparallel β strands, and the loops that connect the β strands cover the open ends of the barrel. The loop between the first two strands is extended with two antiparallel α helices that form a lid-like cover for the open end of the barrel. Inside the barrel, there is a large ligand binding pocket, typical for FABPs [13, 14]. The β barrel is discontinuous, with no main-chain hydrogen bonds present between β strands 4 and 5. This feature is common to the entire FABP family, suggesting structural and/or functional importance.

During myelination, large-scale membrane synthesis takes place, and both protein and lipid components need to be transported to the correct location [15, 16]. P2 could, in addition to being a structural component of the myelin sheath, be important for transporting lipidic compounds into the myelin membrane. For example, myelin is rich in cholesterol [17], and P2 might function as a cholesterol transporter. Studies on P2-deficient mice showed a mild effect on nerve conduction velocity and an abnormal lipid composition during the period of most active myelination, although the ultrastructure of PNS myelin appeared visually normal [4]. A recent follow-up study identified a role for P2 in remyelination after nerve injury [18].

FABPs are small β barrel proteins that can function as lipid transporters [19]. They can roughly be divided into two groups based on their respective transport mechanisms [20, 21]. P2 is a member of the group employing collisional transfer upon interaction with a lipid membrane. The other group includes e.g. liver FABP, having a diffusive transfer mechanism, whereby the FABP spontaneously opens and closes to allow ligand exchange [20]. P2 is unique in the FABP family, as it apparently has a stable contact to membranes, and it can stack lipid bilayers into highly ordered multilayers [6, 12]. Dynamics of the so-called portal region, consisting of helix α2 and the loops β3-β4 and β5-β6, have been identified as a key determinant of ligand binding-related conformational changes in the FABP family, although differences in this behaviour are apparent between family members [22, 23].

Here, we specifically focused on the conserved FABP family residue Phe57 in human P2. Phe57 is in a central position in the portal region, and it is believed to regulate ligand entry into the internal cavity in FABPs [24]. Mutagenesis of Phe57 into alanine (F57A) highlighted specific roles for Phe57 in ligand binding and structural integrity of the portal region. Stability and membrane binding of P2 were affected by the F57A mutation similarly to the CMT-linked point mutations. Furthermore, molecular dynamics (MD) simulations of the F57A mutant showed opening of the β barrel from the side, which is likely to be a general mechanism for FABP portal region opening. The regulation of this conformational change appears to be affected in mutant variants of P2.

## Methods

### Protein production and crystallisation

Wild-type P2 (wt-P2), as well as the F57A and P38G variants, were produced as earlier described [14, 25, 26]. Briefly, the proteins were expressed as His-tagged fusion proteins in *E. coli* using autoinduction [27], and they were purified with immobilized metal ion affinity chromatography (IMAC) and size exclusion chromatography (SEC). The His tag was cleaved with TEV protease, and IMAC and SEC were further used to obtain highly pure protein. Crystallization of the F57A mutant has been described [26]. Two additional crystal forms were obtained here; all crystallization conditions were similar, containing 40–42% PEG6000 at pH 6.0–7.0 (see results for details). Crystallization was done at + 4 °C.

### Crystallographic data collection and structure refinement

Diffraction data were collected on the EMBL beamline X12 at the DORIS synchrotron storage ring (DESY, Hamburg). 20% PEG200 was used for cryoprotection, prior to transferring the crystals to liquid nitrogen temperature. Data were collected at 100 K. The data were processed with XDS [28] and XDSi [29]. The structures were solved with molecular replacement using PHASER [30], using the wild-type P2 structure [14] as template, and refinement was carried out in phenix.refine [31]. Model building was done in coot [32] and validation in MolProbity [33]. The structures were deposited at the PDB with entry codes 6EW2, 6EW4, and 6EW5.

### Lipid preparation

For stock solutions, dimyristoylphosphatidylcholine (DMPC) and dimyristoylphosphatidylglycerol (DMPG) (Larodan Fine Chemicals AB, Malmö, Sweden) were dissolved in chloroform:methanol (9:1 *v*/v) at 10–40 mg/ml final concentrations, and combined to reach an equimolar mixture (DMPC:DMPG (1:1)). Stock solutions and equimolar mixes of dioleoylphosphatidylcholine (DOPC) and dioleoylphosphatidylserine (DOPS) (Avanti Polar Lipids, Alabaster, Alabama) were prepared similarly, but without methanol. The mixtures were divided into aliquots, which were dried under a nitrogen stream at ambient temperature, followed by lyophilization overnight at − 52 °C under vacuum. The dried lipids were stored at − 20 °C until further use. To prepare lipid vesicles, 20 mM HEPES, 150 mM NaCl, pH 7.5 was added directly onto the dried lipids to obtain 5–7 mM concentration. Gentle agitation was applied overnight at ambient temperature. Large multilamellar vesicles (MLVs) were prepared from the suspensions by freeze-thawing 7 times using liquid nitrogen and a warm water bath, followed by vigorous vortexing. Large unilamellar vesicles (LUVs) were prepared by passing the MLVs through a 100-nm membrane 11 times, while heating to + 40 °C. All vesicle preparations were used immediately in downstream experiments.

### Circular dichroism spectroscopy

A Chirascan Plus instrument (Applied Photophysics) was used for circular dichroism (CD) spectroscopy. CD spectra were measured at + 20 °C in a 0.5-mm quartz cuvette. Protein concentration was 0.25 mg/ml (14 μM) in 0.8 mM HEPES pH 7.5, 6 mM NaCl, 0.4% glycerol. In mixtures with lipids, the lipids (DMPC/DMPG at 1:1 mixture) were in 100-fold molar excess (1.4 mM). Temperature scans, at a heating rate of 1 °C/min, were run for the same samples between + 20 − + 90 °C. Full spectra were continuously measured as a function of temperature. Temperature scan data were analyzed using Global3™ (Applied Photophysics).

### Atomistic molecular dynamics simulations

The crystal structure of P2-F57A was subjected to atomistic MD simulations exactly as previously described [11, 25] for wt-P2 and P2-P38G. Simulations were carried out in the presence and absence of bound palmitate, in a simulation box filled with TIP3P water molecules. Briefly, the system was energy-minimized with the steepest descent algorithm and then simulated for a total of 3 μs. MD simulations were run with GROMACS 4.6.7 [34]. The last 2.5 μs were used for analyses. Dynamic cross-correlation map (DCCM) analysis was performed using Bio3D [35].

### Differential scanning calorimetry

Differential scanning calorimetry (DSC) was carried out essentially as earlier described for myelin basic protein (MBP) [36]. P2 and P2 F57A were mixed with MLVs of 350 μM DMPC:DMPG (1:1) in 20 mM HEPES, 150 mM NaCl, pH 7.5, at 1:100 and 1:200 protein-to-lipid (P/L) ratios. The samples were degassed for 10 min under vacuum with stirring at + 10 °C before measurements. A control lipid sample without protein was also prepared.

DSC measurements were performed with a MicroCal VP-DSC instrument. Cell volume was 527.4 μl. The reference cell was filled with 20 mM HEPES, 150 mM NaCl, pH 7.5. Each sample was scanned from + 10 to + 40 °C and back to + 10 °C in 1 °C/min steps. Buffer baselines were subtracted from sample curves and zeroed between + 28–30 °C. All samples were prepared and measured twice and deemed reproducible.

### Membrane surface binding

Surface plasmon resonance (SPR) was performed as described [11, 36]. Briefly, SPR measurements were performed using a Biacore T200 system (GE Healthcare) using 20 mM HEPES, 150 mM NaCl, pH 7.5 as running buffer at + 30 °C. LUVs of 1 mM DOPC:DOPS were immobilized on an L1 sensor chip (GE Healthcare), followed by a blocking injection of 0.2 mg/ml BSA, and a single injection of P2 or P2 F57A. Chip regeneration was performed using a 2:3 (v:v) mixture of isopropanol and 50 mM NaOH. The studied protein concentrations were 0.1–15 μM. A single concentration was analyzed per cycle, and all samples were measured in duplicate. Equilibrium binding responses were plotted against protein concentration and fitted using a one-site binding model (Y = *B*_max_*X / (*K*_d_ + X)).

### Lipid vesicle aggregation

Turbidimetry was used to follow vesicle aggregation, a sign of lipid membrane stacking by P2, as described before [11, 12, 37]. LUVs of 500 μM DMPC:DMPG (1:1) were mixed with 0.5–10 μM P2 or P2 F57A in 100-μl triplicate samples. Turbidity at 450 nm and 660 nm was recorded immediately after sample preparation and mixing at + 30 °C using a Tecan SPARK 20 M plate reader for 15 min. The mixtures were stored overnight at + 4 °C for further co-sedimentation analysis.

### Protein-lipid co-sedimentation

To check for protein co-sedimentation with the lipid vesicles, samples were picked from the 10 μM vesicle aggregation samples, combined, and centrifuged at 16000 g, + 4 °C for 10 min to separate the lipid-bound protein from soluble protein. The pellet was resuspended in 20 mM HEPES, 150 mM NaCl, pH 7.5. The protein content of the supernatant and the pellet suspension was analyzed using SDS-PAGE.

In addition, co-sedimentation assays were separately carried out using 20 μM wt-P2 mixed with 1 mM DMPC:DMPG (1:1) or DOPC:DOPS in 20 mM Tris-HCl (pH 7.5), 150 mM NaCl, 0.5 mM TCEP. After a 30-min incubation at ambient temperature, the samples were subjected to ultracentrifugation for 75 min at 435000 g at + 20 °C. The supernatant and pellet were analyzed by SDS-PAGE.

### Bioinformatics

Using the crystal structure of wt-P2 [14] and all the six independent F57A crystal structures described here, residue interaction networks (RINs) were generated with Chimera [38] through the structureViz [39] app for CytoScape [40], using default parameters without hydrogen bonds, and with own contact detection software (2.5–5 Å). RIN analyses were performed with the RINspector [41] app for CytoScape. They included betweenness and residue centrality analysis. Only Z scores of ≥2 were considered relevant. The analyses were carried out with and without the bound palmitate ligand; the ligand dominated the networks when present. Sequence-based predictions of protein flexibility were done using DynaMine [42, 43]. Structure-based prediction of point mutation effects on P2 thermal stability was done with Cupsat [44] and MAESTRO [45].

## Results

### Myelin P2 folding and stability

**Fig. 1:**
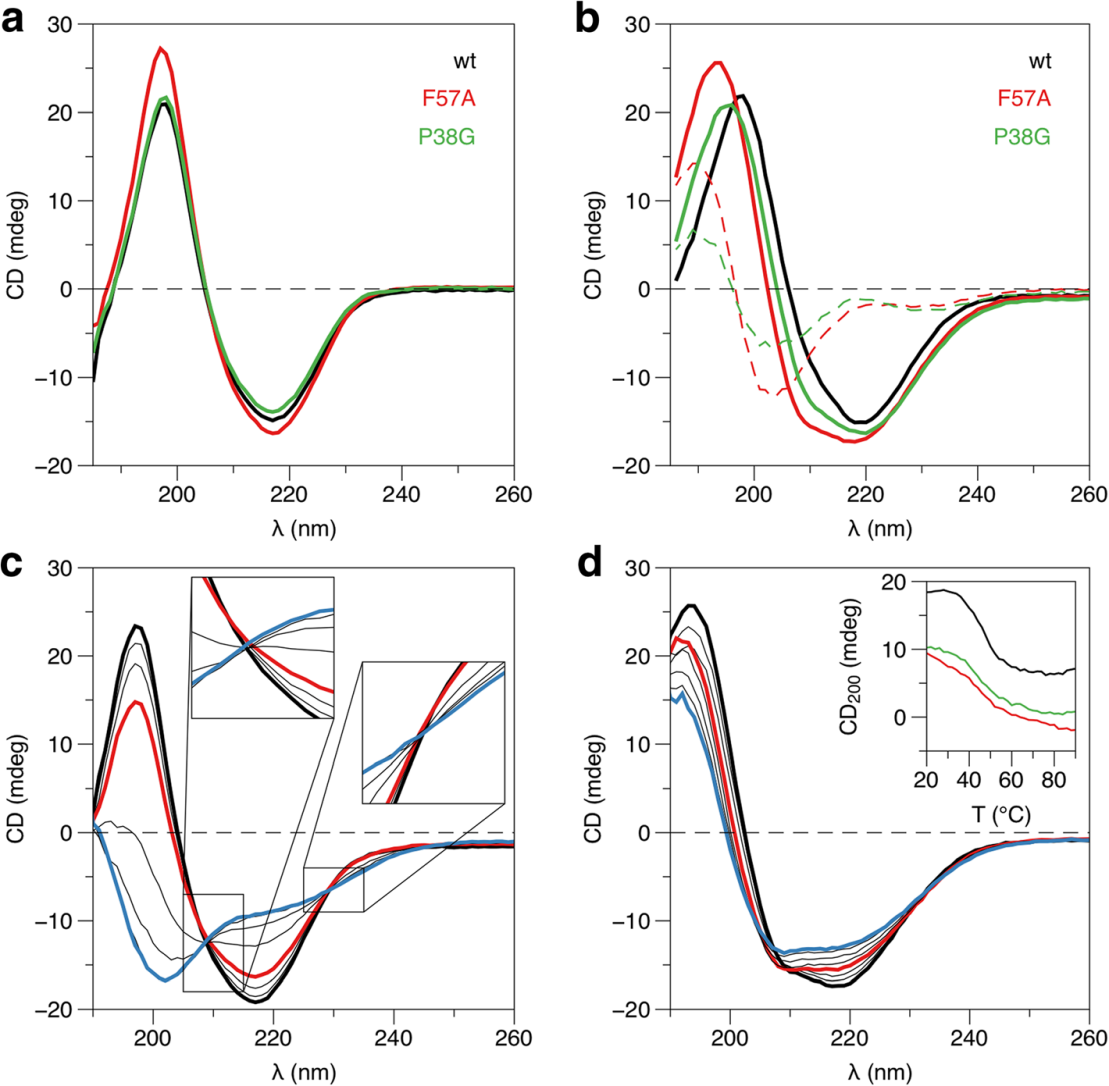
Analysis of P2 folding by CD spectroscopy. **a** Comparison of CD spectra of wt-P2, P2-F57A, and P2-P38G. The spectra for wt-P2 and P2-P38G were presented earlier [25]. **b** Spectra for the same P2 variants in the presence of lipids. The dashed lines indicate difference spectra between wt-P2 and each mutant. **c** Stability analysis of P2-F57A by CD in solution. CD spectra are shown from + 20 (black) to + 90 °C (blue), at intervals of 10 °C for clarity. The spectrum at + 50 °C is shown in red to aid in comparison between samples. **d** Stability of P2-F57A in the presence of lipids. Inset: CD signal at 200 nm of wt-P2 (black), F57A (red), and P38G (green) bound to lipids as a function of T

The F57A and P38G mutants were compared to wt-P2 using CD spectroscopy (Fig. 1a). In solution, all three variants had similar CD spectra, indicating no large-scale structural effects by the mutations. This was earlier confirmed for the P38G mutant [25]. A large effect on the CD spectra of membrane-bound P2 was induced by both mutations (Fig. 1b), and difference spectra indicate that the mutant variants contain less overall secondary structure than wt-P2.

Thermal CD scans were further employed to follow effects of the two mutations on P2 heat stability. In our previous study, we saw a decrease in stability for the P38G mutant [25]. Here, a lowered melting point of the P2-F57A protein was observed compared to wt-P2.

The secondary structure content of P2 changes once mixed with DMPC:DMPG vesicles (Fig. 1b). The temperature scans were repeated with proteins in a lipid environment: wtP2 did not get fully denatured even at + 90 °C, only a conformational shift to a more helical structure was observed [12]. The same behaviour, but including more unfolding, was observed for P2-F57A (Fig. 1c,d).

The different behaviour of wild-type and mutant P2 in solution and in the presence of lipid vesicles shows a strong effect on P2 stability and properties by lipid membrane binding. In solution, both wt-P2 and the mutants show very well-defined isosbestic points (Fig. 1c), indicative of a simple two-state unfolding reaction; even though the melting temperatures are different. However, in the presence of membranes, the spectral changes are gradual, and isosbestic points are not present at all (Fig. 1d). This is a sign of a complex stepwise conformational adaptation to the membrane environment by P2 as a function of temperature. A main change occurs in the range of + 50 °C, and actually at a lower temperature for wt-P2 (Fig. 1d, inset). After this transition, wt-P2 remains stable, while the mutants gradually change their structure even as temperatures approach + 90 °C; however, all the proteins remain in a folded state even at these high temperatures in the presence of lipids. Interestingly, the conformational changes start to be visible around physiological temperature.

### Membrane binding and stacking by P2-F57A

**Fig. 2:**
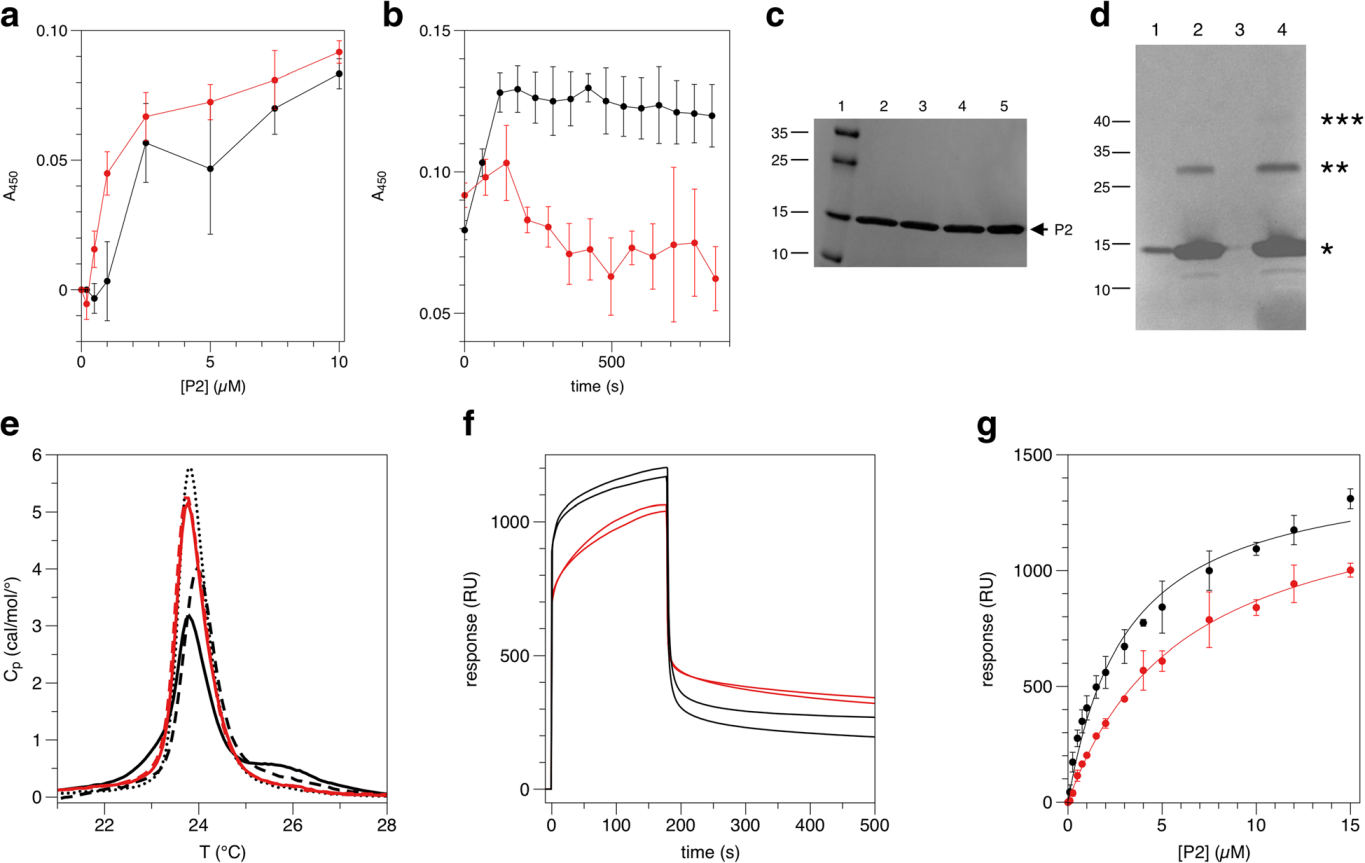
Lipid binding experiments. **a** wt-P2 (black) and P2-F57A (red) initial vesicle aggregation profiles as a function of protein concentration. **b** Decrease in the turbidity signal over time in the mutant sample. **c** Co-sedimentation at 1:100 P/L (10 μM protein with 1 mM DMPC:DMPG) suggests roughly 50% binding of protein to vesicles. 1, molecular weight marker; 2, wt-P2 in pellet; 3, P2-F57A in pellet; 4, wt-P2 supernatant; 5, P2-F57A supernatant. **d** Co-sedimentation of wt-P2 with lipid vesicles. 1–2, supernatant and pellet of 20 μM P2 with 1 mM DMPC:DMPG; 3–4 supernatant and pellet of 20 μM P2 with 1 mM DOPC:DOPS. The asterisks indicate the positions of monomeric (*), dimeric (**), and trimeric (***) P2 in the proteolipid pellet. **e** DSC measurements show the diminished effect of the F57A mutant (red) on the lipid phase transition behaviour, while wt-P2 (black) has a clear effect. Dotted line: lipids alone, dashed line: P/L 1:200, solid line P/L 1:100. **f** Examples of SPR sensorgrams; shown are duplicate injections of 10 μM wt-P2 (black) and P2-F57A (red) onto immobilized DOPC:DOPS (1:1). **g** The steady-state affinity of the F57A mutants to DOPC:DOPS (1:1) vesicles is marginally weaker compared to wild type. All error bars are standard deviations

When P2 is mixed with lipid vesicles, visible aggregation occurs, which can be quantified using turbidimetry [11, 12]. Turbidity can be used as a means to quantify the function of myelin proteins - binding lipid membranes together [36, 37]. At time point 0 of the vesicle aggregation experiments, there is only a marginal difference between P2 and the mutant, when the P/L ratio changes (Fig. 2a). As a function of time, it seems that the proteolipid aggregate stability is higher for wt-P2 (Fig. 2b). F57A reaches its maximum level quicker, but also decreases over time. This suggests differences in vesicle aggregation kinetics between wt-P2 and P2-F57A, which may be linked to effects on the P2 membrane binding mechanism by the mutation. The co-sedimentation of wt-P2 and F57A with lipids is similar (Fig. 2c). Electrophoretic analysis of the proteolipid pellet also occasionally reveals a ladder of different oligomeric forms of P2 in the protein-lipid pellet (Fig. 2d).

Using DSC, we followed the lipid tail phase transition of DMPC:DMPG vesicles in the presence and absence of P2 (Fig. 2e). The lipid phase transition behaviour is altered by P2, and upon addition of wt-P2, the main transition population decreases and a wide, fairly low-intensity population at + 25.5 °C is formed. The effect is dependent on P2 concentration. Earlier, we showed similar effects by MBP on negatively charged vesicles [36]. However, the F57A mutant does not show this behaviour, and the phase transition is not much affected.

We further studied the affinity of P2 towards DOPC:DOPS membranes using SPR (Fig. 2f,g). The mutant seems to have a slightly lowered affinity for DOPC:DOPS (1:1) LUVs (Table 1), while the maximum amount of protein bound to the surface is similar. In addition, as seen before for the CMT disease mutant variants [11], the binding kinetics for F57A are different from wt-P2; apparently both binding and dissociation occur more slowly with the mutant protein.

**Table 1:** SPR fitting parameters of P2 binding to DOPC:DOPS (1:1) vesicles

Protein	*B*_*max*_ (R.U.)	*K*_d_ (μM)	*R* ^2^
P2	1466 ± 54	3.1 ± 0.3	0.9750
P2-F57A	1408 ± 56	6.2 ± 0.5	0.9884

### Structure of P2 F57A mutant in the crystal state

**Fig. 3:**
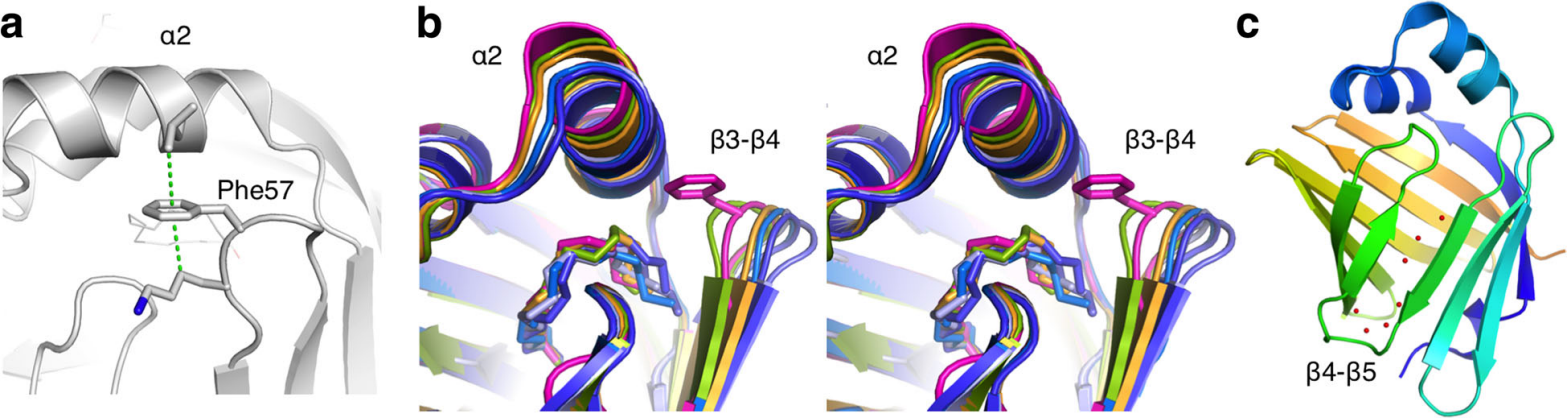
Phe57 in the P2 crystal structure. **a** The location and interactions of Phe57 in wt-P2. Note the C-H...π bonds on both faces of the Phe57 side chain. **b** Conformational effects of the F57A mutation (stereo view). Superposed are wt-P2 (magenta) and all individual P2-F57A monomers from the three crystal forms (6 in total). Note the apparent flexibility of helix α2 and the β3-β4 loop. **c** Discontinuity of the β barrel of P2; a line of water molecules mediates contact between strands β4 and β5

P2-F57A was crystallized in three different crystal forms under similar conditions (Table 2), and the crystal structure was solved from all of them. Phe57 lies at the tip of the β3-β4 loop, close to the bound fatty acid. The fatty acid, originating from the *E. coli* expression host, was modelled as palmitate. Phe57 forms C-H...π interactions with the nearby residues Lys58 and Leu32 that lie on opposite sides of the Phe ring (Fig. 3a).

**Table 2:** Data processing and structure refinement statistics for P2-F57A

Space group	P4_3_2_1_2	C2 [26]	P2_1_2_1_2_1_
Unit cell dimensions	a = b = 58.61 Å, c = 76.96 Å α = β = γ = 90°	a = 112.76 Å, b = 36.08 Å, c = 31.17 Å, α = γ = 90°, β = 96.87°	a = 52.12 Å, b = 76.11 Å, c = 138.43 Å, α = β = γ = 90°
Wavelength (Å)	1.10	1.10	1.10
Resolution range (Å)	20–1.59 (1.63–1.59)	20–1.27 (1.30–1.27)	20–1.95 (2.00–1.95)
<I/σ(I)>	19.1 (1.0)	8.2 (1.2)	11.6 (2.3)
R_sym_ (%)	6.5 (111.4)	7.6 (60.7)	15.8 (75.0)
R_meas_ (%)	7.2 (129.6)	9.2 (78.1)	17.3 (82.1)
Completeness (%)	98.7 (92.7)	96.8 (77.4)	99.1 (98.7)
Redundancy	5.7 (3.2)	2.9 (1.8)	6.0 (6.0)
CC_1/2_ (%)	99.9 (39.9)	99.6 (43.6)	99.4 (75.4)
Wilson B factor (Å^2^)	27.2	16.9	20.2
R_cryst_ (%)	18.6	17.1	18.8
R_free_ (%)	21.6	22.4	23.6
rmsd bond lengths (Å)	0.012	0.009	0.005
rmsd bond angles (°)	1.3	1.2	0.9
Ramachandran favoured/allowed (%)	100/100	99.3/100	98.1/100
# molecules in asymmetric unit	1	1	4
MolProbity score (percentile)	1.25 (97^th^)	1.48 (79^th^)	1.32 (99^th^)
PDB entry	6EW2	6EW4	6EW5

In line with CD spectroscopy, no large-scale changes in P2 structure were induced by the mutation in the crystal state. Once the C-H...π interaction gets eliminated in P2-F57A, both helix α2 and the β3-β4 loop alter their positions: helix α2 turns inwards towards the palmitate bound in the cavity, and the β3-β4 loop bends outwards, slightly opening the portal region (Fig. 3b). In different crystal forms of P2-F57A, helix α2 and loop β3-β4 are in slightly different conformations. The bound fatty acid adopts a more elongated conformation in F57A-P2 than in wild-type P2, correlating with the conformation of the β3-β4 loop (Fig. 3b). Strands β4 and β5 do not have any main-chain H bonds between them, and a line of water molecules mediates contacts between them (Fig. 3c).

### Molecular dynamics simulations

**Fig. 4:**
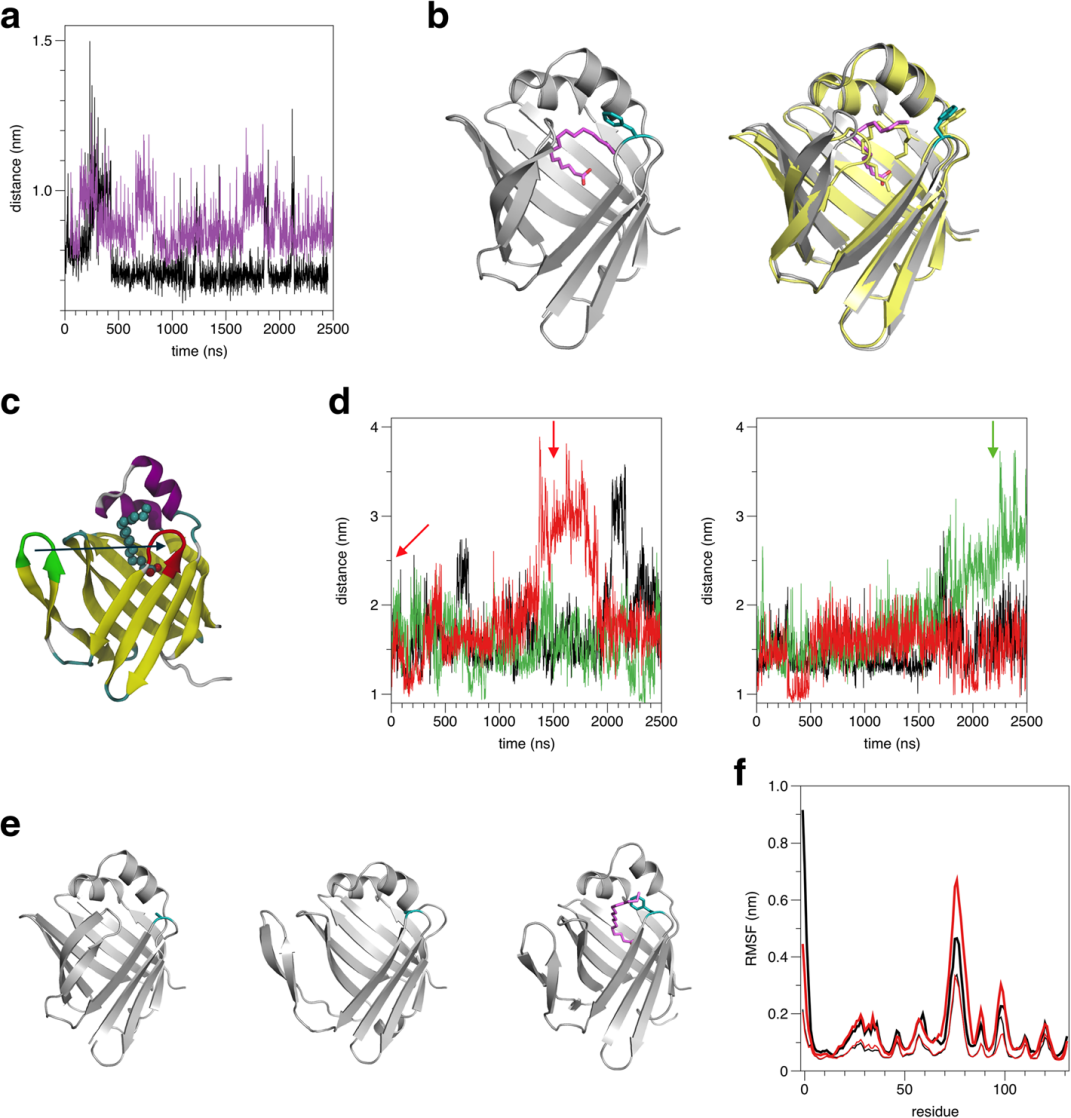
MD simulations of P2-F57A. **a** Fluctuation of the distance between Phe57 and helix α2 shows flipping of the Phe57 side chain in wt-P2 during the simulation. The simulation was run with (magenta) and without (black) the fatty acid ligand. **b** The two conformations of Phe57 (cyan). Left: Phe57 points inwards and interacts with the fatty acid (magenta). Right: Phe57 points outwards. Bovine P2 crystal structure is shown superposed in yellow, with the same conformation. **c** The structure shows the distance measured when studying barrel opening. **d** Distance between the tips of the β3-β4 and β5-β6 loops during the simulation. Black: wt-P2; red: F57A; green: P38G. Left: simulations without ligand. Right: simulations with bound palmitate. The red and green arrows indicate the positions of the snapshopt in the next panel. **e** Structural snapshots from the simulations. Left: Closed starting structure for F57A; middle: open F57A structure at 1.5 μs; right: open P38G structure with ligand at 2.2 μs, identified from our earlier trajectories [25]. **f** RMSF of wt-P2 (black) and F57A (red) in the simulations. Thick lines indicate unliganded simulations and thin lines those with bound fatty acid

In atomistic simulations of wt-P2 in solution, the Phe57 side chain alters its conformation occasionally, flipping outwards and pointing towards bulk solvent (Fig. 4a,b). Such conformations have been seen before in crystal structures of FABPs, including P2 [46]. Phe57 side chain flipping is apparently more frequent and lasts a longer time in the presence of bound fatty acid (Fig. 4a).

The F57A simulations presented here revealed a large-scale opening of the β barrel between strands β4 and β5 (Fig. 4c,d,e). Upon this opening, the ligand-binding cavity becomes accessible, as the hairpin unit formed by strands β5 and β6 flaps out. Based on the F57A simulation results, we re-analyzed our earlier simulations of WT and P38G P2 [25], and indeed, for both of them, an occasional opening of the β barrel at the same site is observed, with exposure of the bound ligand (Fig. 4d). This was especially prominent for the palmitate-bound P38G mutant, which we observed to have higher dynamics than the wild-type protein.

An analysis of the root mean-square fluctuations in the MD trajectories reveals that F57A shows higher dynamics than wt-P2 in the absence of fatty acid (Fig. 4f), while dynamics are identical in the presence of bound ligand. Specifically, the site of the F57A mutation is not much affected, but the β5-β6 and β7-β8 loops are more mobile, indicating an increased flexibility of the portal region in the mutant.

**Fig. 5:**
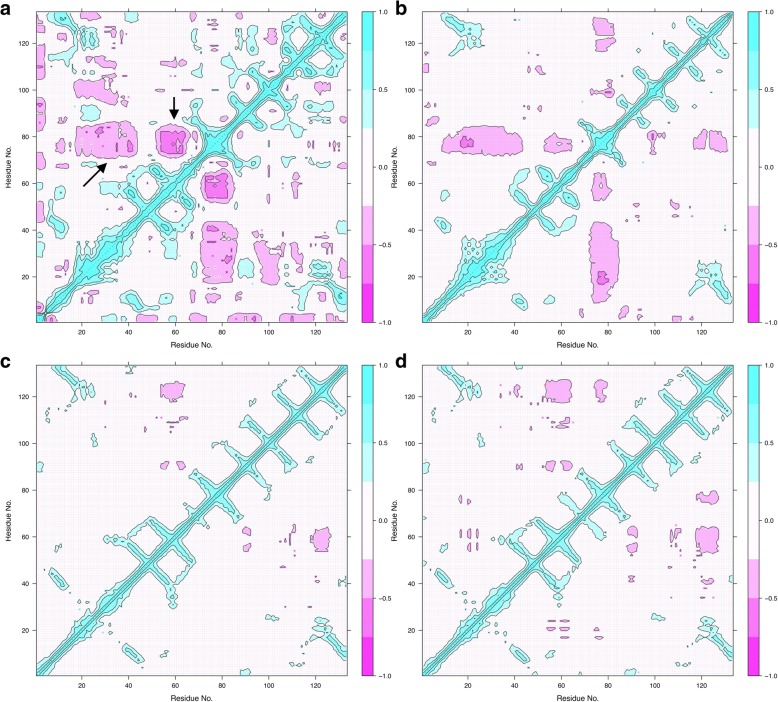
DCCM analysis of MD trajectories. **a** wt-P2 without fatty acid. Note the two regions of strong anti-correlation (arrows). **b** wt-P2 with palmitate. **c** P2-F57A without ligand. **d** P2-F57A bound to palmitate

For additional insight into concerted dynamics of P2, DCCM analyses were performed on the MD trajectories (Fig. 5). While wild-type P2 shows strong anti-correlation between the β5-β6 loop and the rest of the portal region (helices α1-α2 and the β3-β4 loop), no such correlation exists in the F57A mutant. Similar results were obtained with and without bound palmitate (Fig. 5). In addition, we analyzed our earlier MD trajectories for the CMT disease mutations [11] as well as P38G [25]. All the mutations had similar effects to F57A, indicating loss of anti-correlations in portal region dynamics (Additional file 1: Figure S1).

### Predictions and network analyses

**Fig. 6:**
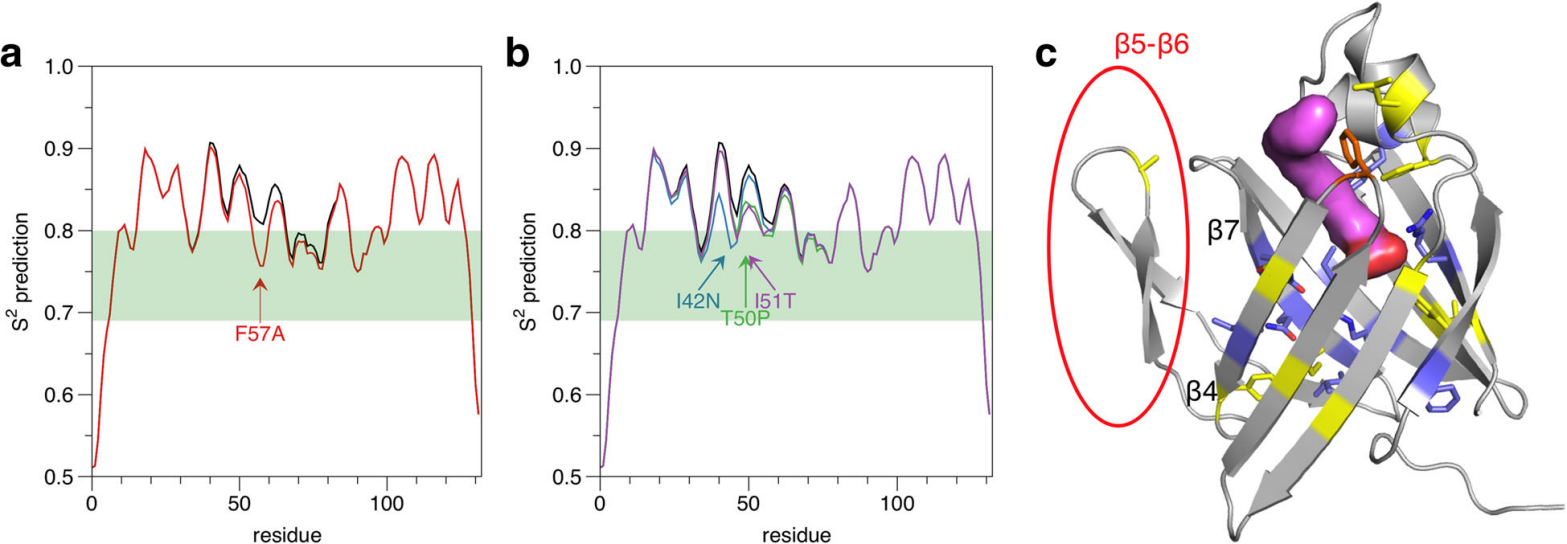
Flexibility and contact network analysis. **a**. DynaMine prediction of F57A flexibility. **b**. DynaMine prediction of flexibility of the CMT disease mutations in P2. Our residue numbering differs by − 1 from the mutation reports, to comply with the conventional numbering of residues in P2 and other FABP structures. **c**. Mapping of central residues onto the P2 structure. The structure shown is the liganded structure of the P38G mutant during MD simulations, to highlight the opening. The centralities were calculated from the unliganded crystal structures. The bound fatty acid is shown as a magenta surface and Phe57 in orange. Residues having high Z scores of centrality are indicated in blue, and the ones showing higher centrality in F57A than the wild-type P2 in yellow. Note how the opening β flap has no residues of high centrality (red circle). See Additional file 1: Figure S1 for more details

Flexibility predictions were carried out based on the P2 sequence, to compare the predicted effects of F57A and the CMT disease mutations. Each of the mutations is predicted to cause a local increase in flexibility (Fig. 6a,b). This is in line with the observed decrease in heat stability and the increased dynamics in simulations for the mutant variants. Based on the P2 crystal structure, we further predicted the effects of the mutations on protein stability (Table 3); overall, the predictions fit the measured decrease of T_m_ for the mutants. This result further shows that the F57A mutation behaves similarly to the disease mutations in structure-function assays.

**Table 3:** Predicted and measured changes in heat stability for various P2 variants. T_m_ values for P38G and the disease mutations have been published [11, 25]

variant	Cupsat ∆∆G (kcal/mol)	MAESTRO ∆∆G (kcal/mol)	Experimental ∆T_m_ (°C)
F57A	−1.91	1.16	−6
P38G	−5.38	1.64	−6
I43N	−7.63	3.92	−17
T51P	−1.51	1.43	−24
I52T	−5.17	2.89	−13

Note that Cupsat defines a destabilizing mutation with a negative ∆∆G, while in MAESTRO, a destabilizing mutation has a positive ∆∆G

For a deeper understanding of P2 folding and intramolecular interactions, residue interaction networks and centralities in the crystal structures were characterized and compared between wt-P2 and P2-F57A; all six independent F57A molecules in the three crystal structures with different space groups were included in the analysis. The analysis shows that Phe57 is structurally not a very central residue in the P2 structure, and its mutation to alanine has subtle effects on these networks (Additional file 2: Figure S2). This observation is slightly unexpected, having experimentally seen a drastic drop in thermal stability in the F57A mutant protein (see above). Interestingly, a group of residues, including the disease mutation sites, as well as residues on strand β4 and in the β5-β6 unit, become more central upon the F57A mutation (Fig. 6c and Additional file 2: Figure S2). Leu32 on helix α2 also belongs to this group. Also the interactions between P2 and the palmitate ligand are slightly altered through the mutation, in line with the subtle conformational changes observed in the respective crystal structures.

When the palmitate ligand is included in the analysis, it dominates the network, highlighting its multiple interactions and suggesting a stabilizing and therefore possible structural role in P2 and the FABP superfamily protein stability (data not shown). The two Arg residues coordinating the palmitate anionic group are central in P2 intramolecular networks, when the palmitate is not taken into account. Additional central P2 residues can be observed. Some of them, especially Gln93 and Gln95, both pointing inwards on strand β7 (Fig. 6c and Additional file 2: Figure S2), have not been of obvious importance in earlier analyses of P2 crystal structures. Gln93 is sandwiched between the charged side chains of Arg106 and Glu72 (close to the β5-β6 loop) - both carrying buried charges inside the protein. Importantly, none of the central residues of wt-P2 lie in the β5-β6 unit, which flips out during barrel opening (Fig. 6c).

## Discussion

While the FABP family contains proteins with distinct functions, current evidence suggests that the FABPs share not only structural, but also dynamical, properties. Phe57 is a highly conserved residue in the FABP family, thought to play an important role in the function of the portal region and ligand binding [24, 47, 48]. Its conformation varies between FABP crystal structures, being either swung in to interact with the hydrophobic ligand and helix α2 or pointing towards the solvent. In structures of P2, Phe57 is normally pointing inwards, making direct van der Waals contacts with the hydrocarbon tail of the bound fatty acid, although the first structure of bovine P2 had Phe57 pointing outwards [46]. We studied the importance of Phe57 in human myelin P2 protein through a variety of biophysical experiments.

### P2 stability and folding

We showed before that binding of wt-P2 to lipid vesicles induced an apparent unfolding of helical structures, i.e. the lid of the β barrel [12]. Hence, we wanted to observe any potential effects on this unfolding by two mutations at the portal region, F57A and P38G. We showed that the P38G mutation renders the P2 protein more dynamic on different time scales [25]. The folding of both of the mutant proteins was affected by the presence of lipid vesicles more than that of the wild-type protein.

Thermal stability of both mutant P2 variants was decreased. For P38G, a flexibility increase in loop areas and helix α2 was also observed in computer simulations [25], and for P2-F57A, the diversity in crystal structures indicates increased protein flexibility in the portal region.

Previously, for wt-P2 it appeared that binding to phospholipids would result in the unfolding of helices [12], whereas the P2 N-terminal peptide was shown to become helical when bound to lipids [14]. It is clear that binding to phospholipids increases P2 thermal stability drastically as a result of an interaction between the phospholipids and P2. This interaction can partially overcome the destabilizing effects of point mutations on the protein in solution. All in all, F57A behaves similarly to both P38G as well as the CMT-linked disease mutations [11, 25].

### Membrane binding and stacking

Many FABPs have been shown to interact with lipid membranes [49–55]. For those FABPs employng a collisional transfer mechanism, such interactions are functionally crucial [49]. To our knowledge, however, P2 is the only member of the FABP family that stacks lipid membranes into multilayers; hence, one can expect its structure-function relationships to differ somewhat from other FABPs.

P2 bound to lipid membrane surfaces, and electrophoretic separation often resulted in a ladder of P2 in the membrane pellet. This reflects a very tight interaction of P2 with stacked membranes, not being dissociated by SDS and heating, and suggests the formation of supramolecular complexes by P2 molecules and two apposing lipid membranes. The structural details of this assembly are likely to be relevant for myelin formation, and further research will be required to elucidate the molecular architecture, also with respect to other myelin proteins present in the same compartment, such as MBP and P0.

Our DSC experiment indicated effects by P2 on lipid phase transition similar to those we observed with MBP [36]. The effect was, however not observed for P2-F57A here*.* This means that the insertion or hydrophobic effect of the protein – or the formation of lipid rafts or structures that have altered phase transition behavior – is not present with the mutant.

### Crystal structures of P2-F57A

Phe57 is a conserved residue in most FABPs, and its stabilizing interactions may be a common feature of FABPs [12]. The conservation concerns especially FABPs thought to transfer ligands to membranes through the collisional mechanism. It is possible that upon membrane binding, these interactions are altered, allowing for conformational changes in the portal region of FABPs.

The possibility of crystallizing in different conformations indicates an increasing flexibility of the portal region, linked to the missing stabilizing C-H…π interactions of Phe57. The absence of Phe57 seems to both destabilize P2 as well as bring about small but clear conformational changes in the crystal state. These differences are likely to correspond to molecular motions upon extrusion of the ligand from the internal cavity. Phe57 lies close to the anion-binding pocket we observed previously [12]. In the structures of the F57A mutant, this pocket is usually occupied by an anionic group in the crystal. We believe this site can be relevant for membrane lipid headgroup binding and possible allosteric conformational changes induced thereby.

While fatty acid binding to FABPs in general causes only little changes in structure and dynamics [23], all experimental structures of P2 thus far have a bound fatty acid molecule inside the protein. Thus, comparisons between liganded and empty P2 cannot be yet done based on experimental data. MD simulations have shown, both in the current study and our earlier work [25], that bound palmitate decreases the overall dynamics of P2.

In all FABPs, the β barrel is in fact discontinuous, and water molecules between two strands interrupt the main-chain H-bonds one would expect for a perfect β barrel. Already since early FABP structures, it has been suggested that this could be a location of FABP flexibility and related to the opening of the portal region [56].

### P2 dynamics and networks

In earlier coarse-grained simulations, Phe57 was found to be one of the residues possibly inserting into the hydrophobic interior of the lipid bilayer [12]. The flexibility of the large Phe57 residue observed in our simulations could be important in the dynamics of the portal region. Phe57 has been considered a gatekeeper of the portal region in FABPs [24, 47, 48].

In the F57A simulation, a large opening of the barrel between strands β4 and β5 was observed. Recently, we observed the same phenomenon in simulations as well as experimental SAXS solution studies for the CMT-linked variants of human P2 [11]. The increased dynamics of the F57A mutant correlate to the opening of the β5-β6 unit. We believe to have observed a general mechanism for opening of the FABP β barrel upon ligand exchange; the helical lid does not open in this process. Earlier studies on different FABPs have identified slightly varying mechanisms for fatty acid entry and egress [22, 57, 58]; while in general, opening of the portal region is considered most important, alternative routes for ligand entry and/or exit have been suggested at the bottom of the β barrel structure [58]. The significant opening of P2 at β5-β6 in our extended 3-μs simulations, which are at least an order of magnitude longer than most published FABP simulations, suggests this is the main ligand entry mechanism in the myelin P2 protein. How the conformational change is affected by binding to lipid membranes, remains currently unknown.

DCCM analyses indicated a clear loss of dynamic anti-correlations between the opening β5-β6 flap and the rest of the portal region in all analyzed mutants (Fig. 5, Additional file 1: Figure S1); similar results were obtained in both the presence and absence of bound fatty acid ligand. In wt-P2, this anti-correlation is likely to reflect coordinated open-close movements, and it appears that such coordination is lost in both the disease variants and the F57A and P38G mutants. These results further highlight the important role of Phe57 in regulating portal region structure and dynamics*.* While Phe57 does not directly interact with the β5-β6 loop, its close interaction with the α2 helix is likely to be important for this indirect regulation; this mechanism may be applicable to also other members of the FABP family.

Similarly to fatty acid binding, also membrane binding mechanisms of various FABPs have been studied [49–55]. The binding was suggested to be heavily affected by macrodipoles present in the protein molecule [53]. The orientation of the FABP with respect to the membrane appears to not be conserved between different FABPs, and docking may occur both through the portal region or the opposite face [53, 59]. In this respect, it is important to remember that P2 is bound between two membrane surfaces, and it is likely that both of these modes are applicable to P2. Our earlier coarse-grained simulations suggested membrane interactions through both the portal region and the anti-portal bottom face of P2 [12].

Gln93 and Gln95, identified in our network analysis, could be crucial for correct folding and function-related conformational changes. Interestingly, alignment of all human FABPs [12] shows that whenever Glu72 is present, also Gln95 is conserved. Residue 93 is in these cases either Gln, His, or Cys; when it is a Cys, residue 106 is Gln instead of Arg. Thus, a residue at position 93 interacts with a buried charge on the β5-β6 unit, and the breaking of this interaction must take place upon β barrel opening. Earlier, it was shown that mutation of Glu72 to Ser caused destabilization of heart FABP [60].

## Conclusions

Phe57 is a residue linking the β-turn loops β3-β4 and β5-β6 and helix α2 in the portal region of P2, and in other FABPs. It interacts directly with the bound fatty acid, and it is likely to play a role in the regulation of portal region opening upon membrane contact. The opening of the β barrel observed in simulations of P2-F57A reveals a mechanism for FABP ligand exchange that has been long suggested [56], but was only recently shown for the CMT disease mutants of P2 [11]. The discontinuity of the FABP β barrel is likely to be instrumental for its opening upon ligand binding - also highlighted by the fact that the hot spot for CMT mutations lies in close vicinity to the opening site in P2, on strands β3 and β4. Comparing all human FABPs, we earlier showed that only two Gly residues are fully conserved in all family members [12]; of these, Gly67 lies in the β4-β5 loop (Fig. 3c), which may act as a functional hinge in the FABP superfamily during barrel opening.

## Additional files

Additional file 1: **Figure S1.** DCCM analyses on earlier MD trajectories from P2 mutants. A. P38G. B. I42N. C. T50P. D. I51T. The empty structures are on the left and the palmitate-bound on the right. (PNG 3329 kb)

Additional file 2: **Figure S2.** Comparison of centrality analyses between wt-P2 and P2-F57A mutants. A residue interaction network (top) was generated from the crystal structures of P2 (bottom), and central residues were mapped onto them. A. Central residues globally conserved between wt-P2 and different P2-F57A mutant structures. Residues are considered central if their Z score ≥ 2, and they are coloured in the network as a function of this Z score with a gradient from yellow (Z score = 2) to red (Z score ≥ 4). Z score values of the wt-P2 were chosen for these Figs. B. Central residues only identified in one or several F57A mutant structures and not in wt-P2. Yellow is indicative of Z score ≥ 2. (PDF 1416 kb)

## Abbreviations

CD: Circular dichroism
CMT: Charcot-Marie-Tooth disease
DCCM: Dynamic cross-correlation map
DMPC: Dimyristoylphosphatidylcholine
DMPG: Dimyristoylphosphatidylglycerol
DOPC: Dioleoylphosphatidylcholine
DOPS: Dioleoylphosphatidylserine
DSC: Differential scanning calorimetry
FABP: Fatty acid binding protein
IMAC: Immobilized metal ion affinity chromatography
LUV: Large unilamellar vesicle
MBP: Myelin basic protein
MD: Molecular dynamics
MLV: Multilamellar vesicle
PNS: Peripheral nervous system
RIN: Residue interaction network
SEC: Size exclusion chromatography
SPR: Surface plasmon resonance
wt-P2: Wild-type P2
