# Supplementary figures and images for: Structure and dynamics of a human myelin protein P2 portal region mutant indicate opening of the β barrel in fatty acid binding proteins
Source: BMC Struct Biol. 2018 Jun 25;18:8. doi: 10.1186/s12900-018-0087-2 (PMC6020228; doi:10.1186/s12900-018-0087-2)

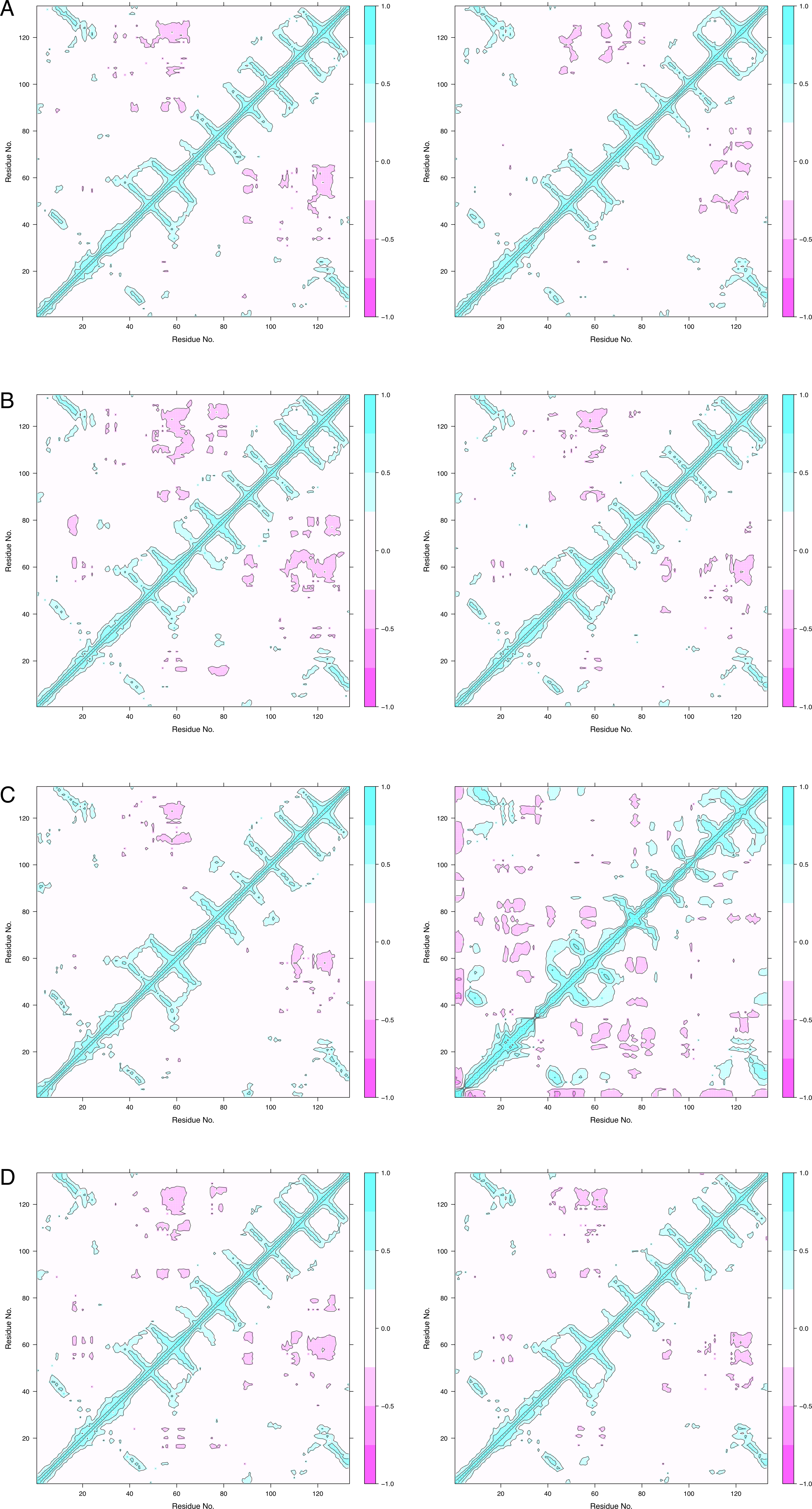

Supplement: Supplementary file 1 — Figure S1. DCCM analyses on earlier MD trajectories from P2 mutants. A. P38G. B. I42N. C. T50P. D. I51T. The empty structures are on the left and the palmitate-bound on the right. (PNG 3329 kb) [file 12900_2018_87_MOESM1_ESM.png]

A

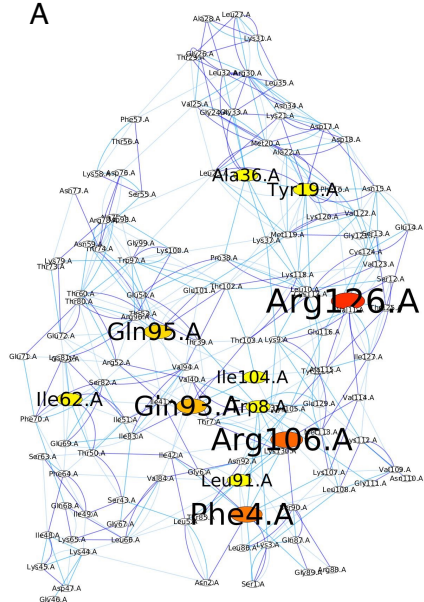

B

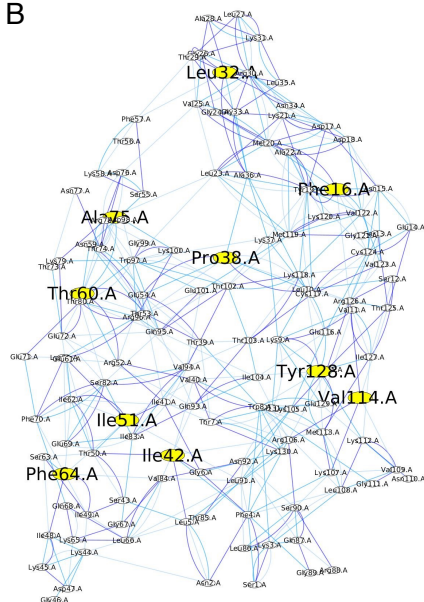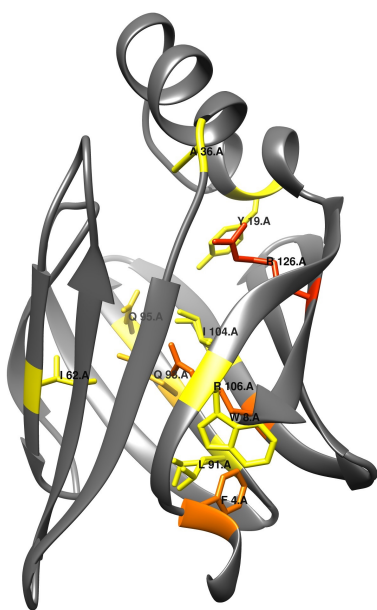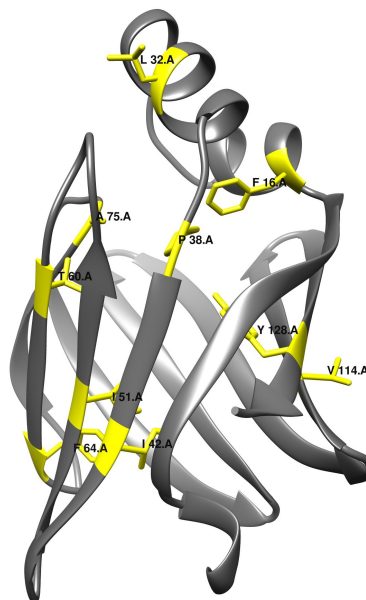

Supplement: Supplementary file 2 — Figure S2. Comparison of centrality analyses between wt-P2 and P2-F57A mutants. A residue interaction network (top) was generated from the crystal structures of P2 (bottom), and central residues were mapped onto them. A. Central residues globally conserved between wt-P2 and different P2-F57A mutant structures. Residues are considered central if their Z score ≥ 2, and they are coloured in the network as a function of this Z score with a gradient from yellow (Z score = 2) to red (Z score ≥ 4). Z score values of the wt-P2 were chosen for these Figs. B. Central residues only identified in one or several F57A mutant structures and not in wt-P2. Yellow is indicative of Z score ≥ 2. (PDF 1416 kb) [file 12900_2018_87_MOESM2_ESM.pdf]
